# Photothermal Effects and Applications of Polydimethylsiloxane Membranes with Carbon Nanoparticles

**DOI:** 10.3390/polym8040084

**Published:** 2016-03-23

**Authors:** Reinher Pimentel-Domínguez, Amado M. Velázquez-Benítez, J. Rodrigo Vélez-Cordero, Mathieu Hautefeuille, Francisco Sánchez-Arévalo, Juan Hernández-Cordero

**Affiliations:** 1 Instituto de Investigaciones en Materiales, Universidad Nacional Autónoma de México, A.P. 70-360, México City 04510, México; postdata.reinher@gmail.com (R.P.-D.); amadovelb@gmail.com (A.M.V.-B.); fsanchez@iim.unam.mx (F.S.-A.); 2 Instituto de Física, Universidad Autónoma de San Luis Potosí, San Luis Potosí, S.L.P. 78290, México; jrvelez@ifisica.uaslp.mx; 3 Cátedras CONACyT, Dirección Adjunta de Desarrollo Científico del CONACyT, México City 03940, México; 4 Facultad de Ciencias, Depto. de Física, UNAM, México City 04510, México; mathieu_h@ciencias.unam.mx

**Keywords:** PDMS-nanocarbon composites 1, photothermal conversion 2, photomechanical actuation 3

## Abstract

The advent of nanotechnology has triggered novel developments and applications for polymer-based membranes with embedded or coated nanoparticles. As an example, interaction of laser radiation with metallic and carbon nanoparticles has shown to provide optically triggered responses in otherwise transparent media. Incorporation of these materials inside polymers has led to generation of plasmonic and photothermal effects through the enhanced optical absorption of these polymer composites. In this work, we focus on the photothermal effects produced in polydimethylsiloxane (PDMS) membranes with embedded carbon nanoparticles via light absorption. Relevant physical parameters of these composites, such as nanoparticle concentration, density, geometry and dimensions, are used to analyze the photothermal features of the membranes. In particular, we analyze the heat generation and conduction in the membranes, showing that different effects can be achieved and controlled depending on the physical and thermal properties of the composite material. Several novel applications of these light responsive membranes are also demonstrated, including low-power laser-assisted micro-patterning and optomechanical deformation. Furthermore, we show that these polymer-nanoparticle composites can also be used as coatings in photonic and microfluidic applications, thereby offering an attractive platform for developing light-activated photonic and optofluidic devices.

## 1. Introduction

A wide variety of synthetic membranes have been developed for applications involving separation processes, leading to useful solutions for wastewater treatments and contaminant removal [1]. Research in polymer membranes has been particularly extensive owing to their simple processing and higher flexibility for hosting other materials [2]. Recent efforts have been devoted to producing reconfigurable materials, in particular, polymers with the ability to adjust specific properties in response to external stimuli such as temperature and electromagnetic radiation [1,2,3]. As an example, changes in the geometry and dipole moment of photoresponsive molecules, such as azobenzenes and triphenylmethane, have proven useful for developing polymer membranes whose wettability and permeability features can be adjusted with light [4]. Other guests commonly used in host polymers include metal nanoparticles, which have shown to yield improved performance of membranes for wastewater treatment [1,2,3]. In general, the inclusion of nanoparticles in polymer membranes aims at improving features such as hydrophobicity and mechanical strength, although new functionalities may also be obtained upon properly combining a polymer host with adequate nanoparticles as guests. Nominally transparent polymers, for instance, may be used as hosts for optically absorbing nanoparticles, thereby providing a new platform for developing photoresponsive polymers.

An interesting photoresponsive material is obtained upon combining polydimethylsiloxane (PDMS) with carbon nanostructures. PDMS has been widely used due to its remarkable thermal, rheological, mechanical and biological properties [5,6,7]. The inclusion of carbon nanoparticles and nanotubes in PDMS membranes has led to obtaining photothermal effects, owing to the enhanced optical absorption observed in carbon-based nanostructures and the subsequent heat dissipation within the host polymer matrix [8,9,10,11]. When irradiated with low power laser light, either focused or guided by an optical fiber, these PDMS-carbon composites show highly localized thermal effects leading to applications such as laser-assisted microfabrication and optomechanical deformation [12,13,14]. This capability for generating highly localized thermal effects can be further exploited in photonics for fabrication, activation, and control of a wide variety of devices. Indeed, recent developments in coating deposition techniques for fiber optic devices have demonstrated that optically controlled tunable devices can be fabricated with PDMS-carbon composites, offering an attractive alternative to electrically activated materials [15]. These coatings can also be used on glass capillaries thereby providing a means to generate optically triggered thermocapillary flow [16], offering novel possibilities for developing optofluidic devices [17].

In this paper, we evaluate the performance of PDMS–carbon composites as photothermal materials for developing polymer membranes and coatings. We focus on their fabrication procedure, and, in particular, on the effects of nanoparticle distribution on their performance as photothermal materials. The composites are based on a PDMS matrix used as a host for carbon nanopowder; these two are mixed following simple procedures yielding membrane samples with different features. Their photothermal properties are evaluated by means of thermal and spectroscopic analyses, showing that the preparation procedure is relevant for obtaining an adequate temperature increase upon irradiating the samples with low power IR laser light. The temperature as a function of laser power is evaluated experimentally and then compared with a theoretical model incorporating the relevant thermal and optical parameters for the PDMS–carbon composites. Heat generation and transfer are evaluated for PDMS samples with embedded carbon nanopowder; furthermore, we also analyze PDMS membranes with a thin layer of carbon nanopowder evenly spread on its surface. While the former composites yield samples with photomechanical features, the irradiated surface of the samples with superficial carbon nanopowder shows a significant increase in temperature adequate for laser patterning. We provide evidence of both phenomena upon direct measurement of the stress induced on the membranes by laser irradiation, and through direct laser patterning on PDMS samples. Finally, we demonstrate a novel method using laser light for inducing two-phase flow in glass capillary tubes coated with the photoresponsive PDMS–carbon composite. We begin by analyzing the mechanisms for heat generation and conduction in polymer-carbon nanoparticle composites, considering relevant parameters such as absorption and thermal conductivity.

## 2. Materials and Methods 

### 2.1. Photothermal Effects in Polymer–Carbon Nanoparticle Composites

Composites such as PDMS with embedded carbon nanoparticles (PDMS/CNPs) fall into the category of what is known as photoresponsive or participating media [9,18,19]. The study of heat generation and transfer in such media constitutes a fascinating subject due in part to its implicit complexity. First of all, the dispersed phase in the composite behaves as scattering centers that deviate the incident radiation in different directions; hence, we are forced to solve not only the global energy equation but also the radiative heat equation, which has an integro-differential form [20,21]. Secondly, the heat transfer and generation parameters, such as the thermal diffusivity and absorption coefficient, change with temperature or are prone to the particular morphology of the dispersion [8]. This necessarily leads to situations where a single CNP concentration may yield distinct heat transfer characteristics depending on how the dispersion was made, or on the functionalization of the CNPs (if any). Despite these and other subtleties found in these kinds of composites, in this section, we intend to enlist some general guidelines governing the heat transfer properties in such systems. In fact, the optical properties of CNPs allow us to employ handy mathematical models for such a highly absorbing media. Indeed, for certain wavelengths and optical intensities, the absorption of PDMS/CNPs composites can be maximized with respect to the scattering effects, reducing the mathematical complexity by decoupling both governing equations leaving the heat transfer process described in terms of a conductive model.

**Figure 1:**
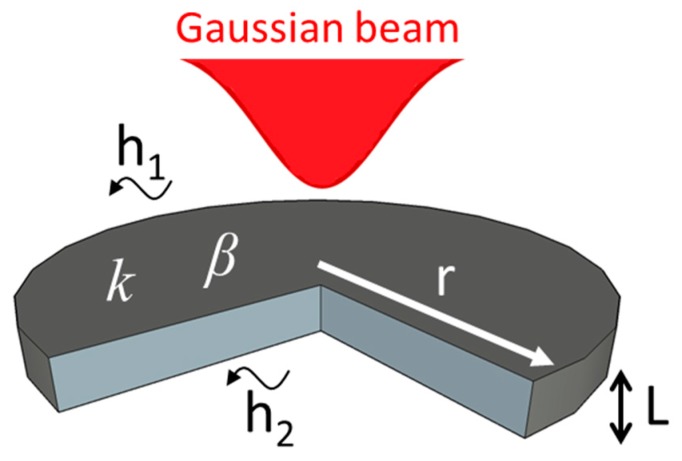
Schematic representation of a composite membrane showing the relevant parameters involved in the mathematical model describing light absorption and heat generation in PDMS/CNP composites (see text).

A simple model to obtain the temperature profile across a membrane (thickness well above the mean free path of the energy carriers) is for the case when the composite is considered as a single continuous material, with an effective absorption coefficient β and thermal conductivity *k* [22]. We therefore assume that β and *k* include the dependence of the two-components properties of the material such as the CNP network morphology, dispersion size, CNP chemical modification and CNPs-PDMS heat interfacial resistance [8,23,24]. In addition, we also assume that the interrogating distances are greater than the mean inter-particle distance. A mathematical model with such assumptions can thus be set as follows [9]: consider a membrane with thickness *L* (see Figure 1) having effective β and *k* parameters and surrounded by dissipating media on both faces (heat transfer coefficients *h*_1_ and *h*_2_). In such a geometry (radial distances much higher than axial distances, *i.e.*, 0≤r≤∞, 0≤z≤L), the increase in temperature ΔT can be computed for a time *t* using Green’s functions in cylindrical coordinates:

(1) $$\Delta T\left(r,z,t\right)=\frac{\alpha }{k}\int_{\tau =0}^{t}\int_{0}^{\infty }\int_{0}^{L}G\left(r,z,t|r\prime ,z\prime ,\tau \right)q\left(r^{\prime },z\prime \right)r^{\prime }dz^{\prime }dr^{\prime }d\tau ,$$

where

(2) $$G\left(r,z,t|r^{\prime },z^{\prime },\tau \right)=\frac{1}{\alpha \left(t-\tau \right)}exp\left[-\frac{r^{2}+r^{2^{\prime }}}{4\alpha \left(t-\tau \right)}\right]{ℐ}_{o}\left[\frac{rr^{\prime }}{2\alpha \left(t-\tau \right)}\right]\times \;\sum_{n=1}^{\infty }\frac{e^{-{\alpha \lambda }_{n}^{2}\left(t-\tau \right)}}{N\left(\lambda_{n}\right)}\left[\lambda_{n}\cos\left(\lambda_{n}z\right)+H_{1}\sin\left(\lambda_{n}z\right)\right]\left[\lambda_{n}\cos\left(\lambda_{n}z^{\prime }\right)+H_{1}\sin\left(\lambda_{n}z^{\prime }\right)\right],$$

(3) $$N\left(\lambda_{n}\right)=\left(\lambda_{n}^{2}+H_{1}^{2}\right)\left(L+\frac{H_{2}}{\lambda_{n}^{2}+H_{2}^{2}}\right)+H_{1},$$

and the values of $\lambda_{n}=\eta_{n}/L$ are given by the transcendental function:

(4) $$\tan\left(\eta_{n}\right)=\frac{\eta_{n}\left(Bi_{1}+Bi_{2}\right)}{\eta_{n}^{2}-Bi_{1}Bi_{2}}\text{ for n}=1,2,3,\ldots$$

In the above equations, α is the effective thermal diffusivity, $H_{1,2}=h_{1,2}/k$ is the ratio between the heat transfer coefficients and the thermal conductivity, and $Bi_{1,2}=H_{1,2\;}L$ is the Biot number. Subscripts 1, 2 correspond to the irradiated and rear surface, respectively. Let us consider two forms for the heat generation term q(r,z) depending on the procedure followed to incorporate the CNPs in the PDMS matrix. We consider as well that heat is generated by the photothermal response of the composite upon irradiation with a laser beam having Gaussian intensity profile (see Figure 1). For CNPs evenly dispersed within the polymeric matrix, the q(r,z) term can be formulated using Beer-Lambert’s law [19,20]:

(5) $$q\left(r,z\right)=\left(1-ℛ\right)\beta I_{o}\text{exp}\left(-\beta z-r^{2}/A\right),$$

where $A=w^{2}/2$ and *w* is the laser beam radius at which the optical intensity $I_{o}=P_{0}/A\pi$ decays to $e^{-2}$; $P_{0}$ is the optical power and R accounts for the non-absorbed incident radiation (e.g., due to surface reflectivity). If the CNPs are deposited on the surface of one side of the membrane (as in the procedure where PDMS is cured over a bed of CNP, see below) the pertinent form of the generation term becomes:

(6) $$q\left(r,z\right)=2\left(1-ℛ\right)I_{o}\delta \left(z\right)\text{exp}\left(-r^{2}/A\right),$$

This accounts for the fact that the radiation is absorbed on a very thin layer of material, *i.e.*,:

(7) $$lim_{\beta \to \infty }\frac{\beta }{2}e^{-\beta z}=\delta \left(z\right),$$

where δ(z) is the delta function. Using this formulation and some integral formulas [9], we can obtain analytical solutions for ΔT at points of interest. For example, the transient ΔT at the point located at the rear surface exactly opposing the beam axis (*r* = 0) impinging on a membrane in which CNP are mixed with the PDMS has the form [9]:

(8) $$\Delta T{\left(L,t\right)}_{axis}=\left(1-ℛ\right)\frac{A\beta I_{o}}{2k}\overset{\infty }{\underset{n=1}{\sum }}\frac{I_{1}\left(\lambda_{n}\right)}{N\left(\lambda_{n}\right)}\left[\lambda_{n}\cos\left(\lambda_{n}L\right)+H_{1}\sin\left(\lambda_{n}L\right)\right]e^{\frac{\lambda_{n}^{2}A}{4}}\;\times \left[Ei\left(-{\alpha \lambda }_{n}^{2}t-\frac{A\lambda_{n}^{2}}{4}\right)-Ei\left(-\frac{A\lambda_{n}^{2}}{4}\right)\right].$$

Here, Ei denotes the exponential integral and

(9) $$I_{1}\left(\lambda_{n}\right)=\frac{1}{\beta^{2}+\lambda_{n}^{2}}\left[\lambda_{n}\left(\beta +H_{1}\right)+e^{-\beta L}\left\{\sin\left(\lambda_{n}L\right)\left(\lambda_{n}^{2}-H_{1}\beta \right)-\lambda_{n}\cos\left(\lambda_{n}L\right)\left(\beta +H_{1}\right)\right\}\right].$$

Similarly, the final expression for the case when CNP cover only the irradiated surface is:

(10) $$\Delta T{\left(L,t\right)}_{axis}=\left(1-ℛ\right)\frac{AI_{o}}{k}\sum_{n=1}^{\infty }\frac{\lambda_{n}}{N\left(\lambda_{n}\right)}\left[\lambda_{n}\cos\left(\lambda_{n}L\right)+H_{1}\sin\left(\lambda_{n}L\right)\right]e^{\frac{\lambda_{n}^{2}A}{4}}\times \;\left[Ei\left(-\alpha \lambda_{n}^{2}t-A\lambda_{n}^{2}/4\right)-Ei\left(-A\lambda_{n}^{2}/4\right)\right].$$

Equations (8) and (10) show that ΔT maintain a linear relation with the optical power ($P_{o}\sim AI_{o})$. Hence, as far as the conduction regime is concerned, ΔT can be predicted for any value of $I_{o}$ once the factor $\Delta T/I_{o}\left(k,\beta ,h_{1,2},L,R,w\right)$ is known. Another interesting feature of the conduction regime is that ΔT is inversely proportional to the thermal conductivity *k*. Thus, membranes with good thermal conductivities will behave more like heat removers rather than heat generators (*i.e*., they will oppose local increases of ΔT). Note also that ΔT increases with the absorption coefficient β, which is an expected behavior, although much less is known about how β depends on the CNP dispersion and/or clustering. Remarkably, the theoretical model predicts that the composites can be designed with different heat generation/dissipation properties due to the opposing dependencies of β and *k* on the dispersion morphology. The effects of CNP clustering on the thermal features of the composites will be explored in the following sections.

A final note should be given concerning the heat transfer coefficients $h_{1,2}$ and their roles when comparing theoretical and experimental results. If the temperature is measured by non-contact devices (e.g., with an IR camera), $h_{1,2}$ are easily known since they must correspond to the value of heat convection in air ($\sim O\left(10\right)\;W/m^{2}K$); however, if ΔT is measured with contact devices (thermocouples, resistive temperature sensors), $h_{2}$ will be larger, and usually unknown because direct contact with the membrane adds an additional dissipation and interfacial resistance [9].

### 2.2. PDMS/CNP Membranes: Materials and Preparation

**Figure 2:**
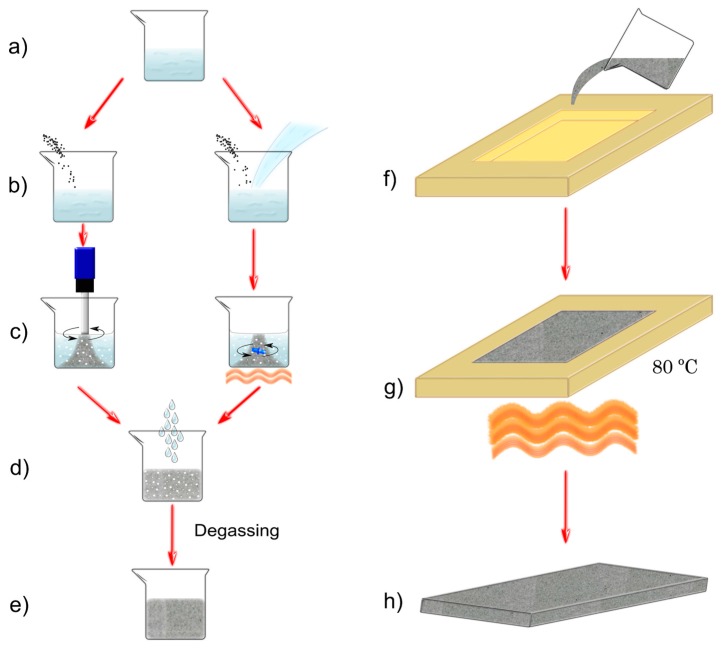
Preparation method for the PDMS/CNP membranes showing two mixing processes to obtain different CNP cluster sizes. (**a**) Weighting of the silicone oil inside of a glass beaker, (**b**) addition of the CNP to the PDMS, (**c**) mixing of the CNP with PDMS (evaporation by heat (orange lines) when CHCl_3_ is used), (**d**) addition of curing reagent, (**e**) degassing of the blends, (**f**) PDMS/CNP mixture poured into a glass mold, (**g**) curing process by heat (orange lines) at 80 °C for solidification, and (**h**) unmolding PDMS/CNP samples.

Membranes of PDMS/CNP composites were fabricated using Dow Corning Sylgard 184 PDMS, with carbon nanopowder (particle size < 100 nm, Sigma Aldrich, St. Louis, MO, USA, CAS: 633100). The use of nanopowder instead of particles with defined geometries allows for minimizing any optical and/or thermal effect attributable to particle geometry. Furthermore, materials with high commercial availability were preferred in order to obtain low-cost materials for fabricating the membranes. Incorporation of the CNP is of crucial importance to generate a uniform distribution in order to yield homogeneous properties along the whole membrane. Two approaches were used when mixing the CNP with the PDMS aiming at obtaining different CNP cluster sizes. We first used a dispersing device (Ultra-Turrax T18, IKA Werke GmbH & Co., Staufen, Germany) for mixing both components of the composite membranes and these were labeled as PDMS+CNP. The second approach involved the use of chloroform during the mixing process, aiming only at reducing the viscosity of the silicone oil of the PDMS. Under this condition, the CNPs are easily incorporated into the polymer matrix by means of simple mixing using a magnetically driven stirrer, avoiding further modification of the materials [10,11,25]. The addition of CHCl_3_ yielded a homogeneous aqueous solution and the composites obtained with this procedure were labeled as PDMS+CNP+CHCl_3_. The fabrication process is illustrated in Figure 2 and involves the following steps:

(a) The required base material of the elastomer (silicone oil) is weighted inside of a glass beaker.
(b) CNP is added in a concentration of 0.1% in weight compared to the total PDMS amount. For the PDMS+CNP mixture, the nanopowder is simply poured into the beaker; alternatively, chloroform is also added in a proportion of 0.75 mL per g of PDMS in order to obtain the mixture for the PDMS+CNP+CHCl_3_ samples.
(c) The materials are mixed to evenly distribute the carbon nanopowder in the silicone oil. For the PDMS+CNP, mixing of the materials is achieved with the dispersing device operating for 5 min at a speed of 2800 rpm, and subsequently increasing the speed to 3600 rpm for 8 additional minutes. The second mixture, PDMS+CNP+CHCl_3_, yields a liquid solution due to the addition of chloroform; this is mixed using a magnetic bar activated by a hot plate with magnetic stirrer while heated at 150 °C until full evaporation of the CHCl_3_.
(d) The curing agent is added with a 1:10 ratio compared to the base material and both parts are mixed by hand during two minutes.
(e) Air captured during the mixing process is removed thru a degassing process.
(f) The PDMS/CNP mixture is poured into a glass mold; this was fabricated from glass substrates and height spacers of approximately 420 μm. The excess of material is then removed using the doctor blade technique.
(g) Solidification of the composites is achieved upon heating at 80 °C during 2 h.
(h) After cooling at room temperature, the resulting PDMS/CNP composite is finally removed from the mold.

**Figure 3:**
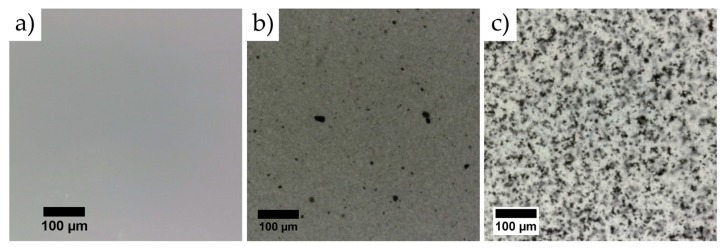
Optical microscopy images of the fabricated membranes: (**a**) pure PDMS; (**b**) PDMS+CNP; and (**c**) PDMS+CNP+CHCl_3_. The magnification is the same for the three images (the scale bar is shown in (**a**)).

Optical microscopy images of the resulting PDMS/CNP membranes are shown in Figure 3; for comparison, we also include an image of a PDMS membrane (*i.e*., without CNP) cured under the same conditions as those followed for the composites. The PDMS+CNP samples show more uniformly distributed CNP within the membrane with only a few carbon clusters separated from each other (Figure 3b). In contrast, a larger amount of clusters in close proximity are apparent in the PDMS+CNP+CHCl_3_ samples (see Figure 3c), owing to a less uniform distribution of CNP forming a percolating network morphology. The membranes obtained with the dispersing device (PDMS+CNP) thus show a more homogeneous distribution of CNP, and with much fewer clusters within the PDMS matrix. As demonstrated in the following sections, these features determine the photothermal performance of the composite membranes.

## 3. Results and Discussion

Several tests were carried out to characterize the composite membranes. We focused on evaluating the effects of the fabrication process on the photothermal, structural and photomechanical features of the membranes.

### 3.1. Photothermal Effects: Heat Generation and Transfer in the Composites

**Figure 4:**
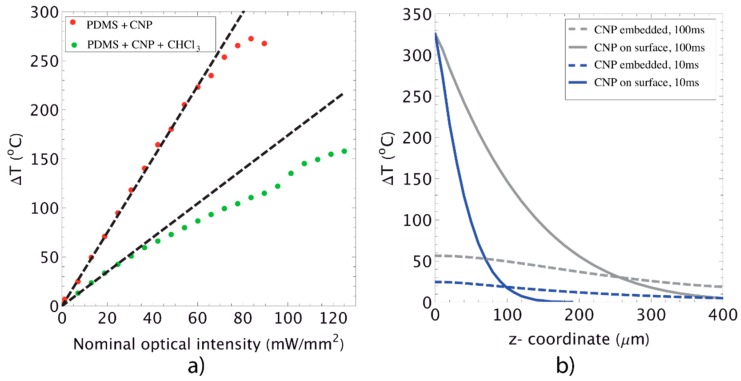
Heat generation and transfer in PDMS/CNP membranes: (**a**) comparison of the experimental ΔT (dots) and theoretical (dashed lines) values using Equation (8) and the parameters given in Table 1. The parameter *n* in Equation (8) was set to 48 and *t* = 1 min, which was the experimental time to attain a steady value. The nominal optical intensities were calculated as $I_{N}=P_{o}/\pi w_{o}^{2}$ , where *w*_o_ is the spot radius estimated by using the NA of the optical fiber and fiber tip-membrane separation (5.82 mm); (**b**) comparison of the theoretical ΔT profiles along the membrane thickness using Equation (8) and (10) for the two CNP dispersion types: one where the CNP are dispersed in all the polymer matrix, and another where the CNP are deposited just on the irradiated surface.

We begin by analyzing the heat generation and transfer features as predicted by the mathematical model obtained in Section 2.1. Figure 4a shows the experimental and theoretical values of *∆T* at the point (*r =* 0*, z = L*) for the PDMS+CNP and PDMS+CNP+CHCl_3_ samples. Heat was generated in the membranes by direct irradiation with a 975 nm laser diode coupled to an optical fiber (Corning SMF-28e, Corning, NY, USA, numerical aperture NA = 0.14); the resulting output beam had a Gaussian profile and was located 5.82 mm away from the membranes. The experimental values of *∆T* were measured using an IR camera (Fluke Ti300, Everett, WA, USA), while the theoretical values were obtained using Equation (8) and the parameters listed in Table 1.

The close agreement between the experimental measurements and the theoretical values at low optical powers confirm that *∆T* is linearly proportional to the optical intensity, thus validating the conduction regime assumption used in the model. Notably, Figure 4a suggests that deviation from the linear response most likely arises from changes in the optical properties of the material (e.g., reflectivity or emissivity at the surfaces) rather than on parameters subject to temperature changes. Another interesting observation is that the linear-conduction regime holds even though optical limiting behaviors (scattering effects) are already present in the membranes. It has been shown that optical limiting effects appear at values generally above 1 J/cm^2^ for similar wavelengths as those employed in this work [21,26]; this value is easily overpassed in our experiments considering that the laser beam is maintained for periods of about one minute. A complete interpretation of such results will demand computation of the radiative heat flux inside the membrane. However, a simple phenomenological description can be given in terms of the absorption/scattering compensation: while scattering increases with the optical intensity $I_{N}$ along with the propagation of light inside the material, the ΔT generated by the newly illuminated volume is compensated by a reduction in the absorption coefficient [21] (recall that the extinction coefficient is the sum of the absorption and scattering terms).

Another important observation can be made regarding the values of β and *k*. The experimental measurements shown in Table 1 indicate that β increases for an improved dispersion of the CNP fillers: while β = 4.4 mm^−1^ for the membranes fabricated with the dispersing device, β = 2.8 mm^−1^ for those using chloroform. Notice, however, that fitting Equation (8) with the experimental data yields a decrease in *k* with the dispersion of CNP (see Table 1), confirming that β and *k* maintain opposite dependences on the composite morphology. As seen in Figure 3, the CNPs are more homogeneously distributed in the PDMS+CNP membranes. In contrast, the PDMS+CNP+CHCl_3_ samples show a large number of clusters of CNP distributed within the sample. While an improved distribution of CNP leads to a larger attenuation coefficient (PDMS+CNP), clusters within the membranes lead to improved thermal conductivity, associated with a percolating network morphology (PDMS+CNP+CHCl_3_). These results can thus be interpreted in terms of the CNP interconnectivity. The PDMS+CNP samples include only a few isolated clusters with low connectivity, yielding a membrane with good thermal generation capabilities but with poor thermal conductivity (good thermal accumulation, low thermal dissipation). On the other hand, the PDMS+CNP+CHCl_3_ samples contain a larger amount of clusters with high interconnectivity, yielding a membrane with poor thermal accumulation but good dispersion qualities. As shown in the following sections, the CNP also increases the crystallinity compared to pristine PDMS. This feature, together with the improved CNP clusters interconnectivity obtained when using chloroform as solvent, provides a plausible explanation of the increase in thermal conductivity obtained in our experiments.

We finish this section comparing the theoretical ΔT profiles inside the membranes made by mixing the CNP with the PDMS and the others having CNP spread at the irradiated surface (see Equation (10)). Figure 4b shows the resulting ΔT profiles obtained along the axial direction using Equations (8) and (10). The parameters used in both equations were again taken from Table 1 from the PDMS+CNP membrane. Two situations have been included in the comparative plot; in one case, both membrane conformations are irradiated with a Gaussian beam ($I_{N}=73.5\;\text{mW}/{\text{mm}}^{2})$ during 100 ms (*i.e*., 0.73 J/cm^2^); in the other case, the membranes are irradiated with $I_{N}=209.8\;\text{mW}/{\text{mm}}^{2}$ during 10 ms (*i.e*., 0.21 J/cm^2^). Clearly, while the membranes with embedded CNP generate, at the most, 20 and 50 °C during these short time periods, the membranes having CNPs spread at the irradiated surface attain a maximum *ΔT* ~ 300 °C at *z = 0*, while, on the rear surface (*z = L*), the temperature is barely increased. Such an increase in temperature corresponds to the flash point of PDMS [27] and allows for laser ablation of the composite. As discussed in the final sections, this feature allows for laser patterning of the composite membranes.

### 3.2. Structural and Mechanical Characterization of the Composites

**Figure 5:**
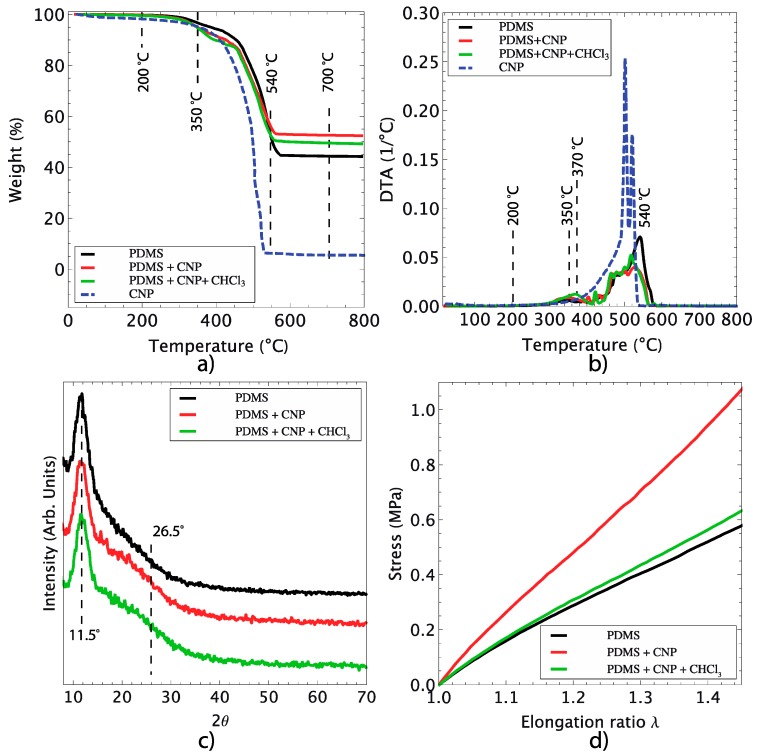
Thermal and mechanical characterization of the composite membranes: (**a**) thermogravimetric analysis of PDMS and its composites; (**b**) derivatives of thermogravimetric analysis evidencing the critical temperatures of degradation for PDMS and its composites; (**c**) X-ray diffraction spectra showing amorphous and crystalline features of PDMS and composites; (**d**) mechanical response of PDMS and composites under uniaxial tensile load.

Thermogravimetric analysis (TGA) provides information about the thermal decomposition of the samples as a function of temperature. The TGA results for samples of CNP, pure PDMS and the PDMS/CNP composites are shown in Figure 5a. Clearly, the three polymer-based samples are completely stable from room temperature (25 °C) and up to temperatures just below 200 °C; beyond this temperature, the thermal degradation of pure PDMS occurred at 350 °C. Notice, however, that the CNP start degrading at even lower temperatures. Upon analyzing the first derivatives of the TGA curves, two stages can be identified at which significant weight loss occurs (Figure 5b). The first one is located between 300 and 400 °C and corresponds to PDMS degradation into cyclic oligomers due to Si–O bond scission [28]. The second stage is observed when the PDMS is exposed to higher temperatures (480 and 626 °C) at a slow heating rate; hence, thermal degradation of PDMS starts via depolymerization of the polysiloxane backbone. This leads to the formation of cyclosiloxanes [29], and it has been shown that a radical mechanism occurs throughout the Si–CH_3_ bond scission yielding methane through hydrogen abstraction [30]. As a consequence, the flexibility of PDMS decreases due to the crosslinking effect of macro-radicals; this, in turn, increases the thermal stability of the sample, and bond reorganization occurs leading to silicon–oxycarbide residues. The thermal degradation of PDMS can be affected by factors such as end-group functionality, impurities or solvent and oxidation. In our case, the addition of a small percentage of CNP (0.1%) slightly reduced the thermal degradation of the composite membranes compared to pristine PDMS samples (around 2%). Notice also that no significant differences are apparent for the thermal degradation of the two composite membranes, occurring between 250 and 300 °C.

X-ray diffraction analysis did not show significant carbon nanoparticle features, owing to the low concentration (0.1% *w/w*) used to fabricate the membranes (see Figure 5c). Nonetheless, the characteristic peaks of PDMS were easily detected, with the most intense peak located at 11.5° corresponding to its tetragonal phase. Only a small change in the diffraction pattern was observed for the composites, occurring at 26.5°, hardly perceptible, and possibly attributable to the inclusion of the CNP. The percentage of crystallinity for the samples was found to be 24% for PDMS, 38.3% for PDMS+CNP, and 38.4% for PDMS+CNP+CHCl_3_. This improvement in crystallinity observed in both samples can be attributed to an arrangement of polymeric chains favored by the CNP acting as nucleation centers yielding crystalline zones. In principle, an increase in crystallinity should improve the thermal stability; however, in our experiments, the composite membranes show thermal degradation at temperatures close to those observed for pristine PDMS. Notice, however, that the CNPs incorporated in the membranes start degrading even at lower temperatures, thus contributing to the registered weight loss during the TGA test. Further weight loss is accounted for by PDMS degradation occurring within the same temperature range.

To evaluate the mechanical behavior of the composites, dog-bone specimens were prepared following the ASTM D1708 standard. These were then evaluated by means of uniaxial tensile experiments, carried out with a custom-designed device [31]. As shown in Figure 5d, the registered stress for PDMS and its composites varies in a nonlinear fashion as a function of the elongation ratio (λ). This is consistent with previous reports on elastomeric composites with carbon-based nanoparticles; from the resulting curves of the uniaxial tests, the shear modulus and Young’s modulus of the membranes can be calculated as reported elsewhere [14]. For comparison purposes, the stress curves shown in Figure 5d are plotted only up to λ *=* 1.4; notice that the inclusion of CNP clearly modifies the mechanical behavior of the samples. In particular, when compared with pure PDMS, the composite membranes show an increase in the shear module, as observed by the higher stresses registered for similar elongation ratios. Furthermore, the curves provide evidence of the influence of the fabrication process on the mechanical behavior of the membranes. The PDMS+CNP showed higher stresses owing to a more homogeneous distribution of CNP clusters within the PDMS matrix (see Figure 3). In contrast, the PDMS+CNP+CHCl_3_ membranes showed only a slight improvement when compared to pure PDMS. Although the crystallinity of PDMS+CNP and PDMS+CNP+CHCl_3_ were almost the same, the mechanical behavior of the composites was different. In particular, the shear modulus of PDMS+CNP was estimated to be 0.87 MPa, larger than that of the PDMS+CNP+CHCl_3_ membrane estimated as 0.58 MPa. Meanwhile, the pristine PDMS membrane resulted in a 0.55 MPa shear module. These results suggest that the composite prepared by mechanical stirring at higher RPMs (Ultra-Turrax T18 mixing device, IKA Werke GmbH & Co.) effectively reduces the size of the carbon clusters (see Figure 3b). This in turn favors the incorporation of the carbon particles within the polymer network, thus reinforcing the polymer matrix. In contrast, the large number of CNP clusters observed in the PDMS+CNP+CHCl_3_ membranes seems to be acting as inclusions favoring stress concentration without increasing the strength significantly.

### 3.3. Photomechanical Effects: Light Induced Stress

**Figure 6:**
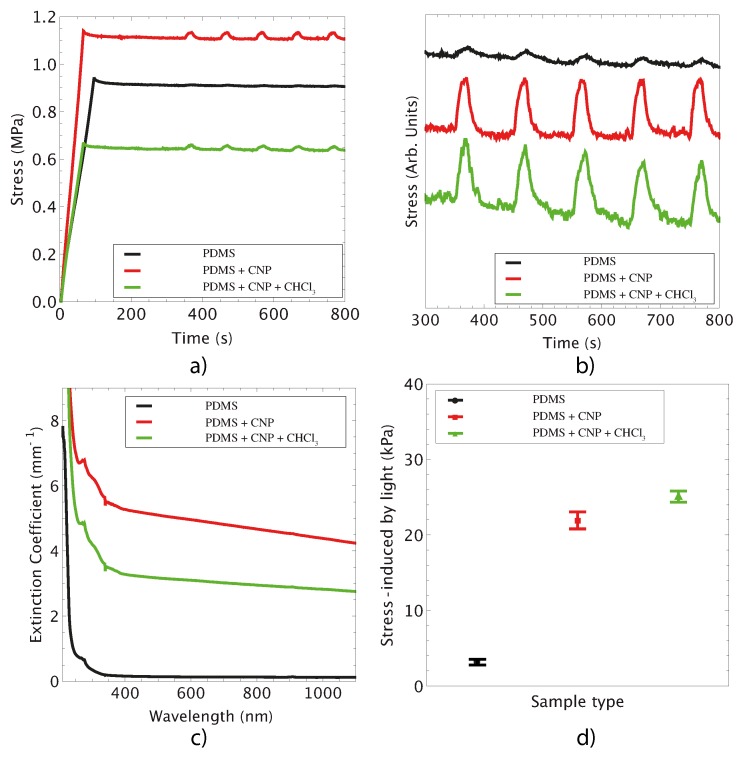
Laser induced mechanical stress in PDMS composites. (**a**) experimental curves showing the stress relaxation as function of time for PDMS and its composites; (**b**) peaks evidencing the photo-actuation capabilities of these composites; (**c**) extinction coefficient as a function of wavelength for PDMS and composites; and (**d**) photomechanical actuation of PDMS and composites.

To evaluate the photomechanical response of the membranes, the samples were subjected to a stress relaxation test and irradiated with laser light. The test was performed for elongation ratios of 1.4 < λ < 1.7, and laser irradiation was delivered via the infrared (IR) laser diode. Figure 6a shows the stress relaxation curves registered for the samples during the test; noticeably, laser irradiation induces an increase in stress in the samples. Further evidence of this effect is provided in Figure 6b, showing the stress registered in each sample for five pulses of the IR laser (optical power of 136 mW). This effect induced by laser light is due to optical absorption of the CNP and subsequent heat release within the polymer matrix [11,12,13,14]. The inclusion of CNP in PDMS thus provides a means to obtain photothermal effects in an otherwise transparent material. The increase in stress in the samples is explained upon considering the PDMS and its composites as systems whose entropy is reduced when they are pre-stretched in the relaxation test. Pre-stretching causes a reduction in entropy because the polymeric chains tend to align along the direction of the applied force. Hence, when the IR laser is turned on, and after some characteristic time (20 s for the tested samples), the photothermal effect generates a highly localized increase in temperature yielding heat percolation within the polymeric matrix. This leads to an increase in entropy and the polymeric chains that were previously aligned during the pre-stretching recover their initial state. Globally, this set of events produce mechanical contraction of the sample, and this is registered by the load cell and graphically represented by a peak in the stress relaxation curve (see Figure 6b). Similar behavior has been observed in graphene/elastomer composites [32,33] and PDMS doped with carbon soot particles [14].

Measurements of the extinction coefficient (Figure 6c) clearly show that pristine PDMS is optically transparent to IR irradiation, while PDMS+CNP and PDMS+CNP+CHCl_3_ indeed show extinction coefficients arising from absorption and scattering due to the inclusion of CNP. Notice that the membranes prepared with CHCl_3_ had a lower extinction coefficient compared to that obtained for the membranes prepared with the dispersing device. In contrast, as shown in Table 1, the use of CHCl_3_ during membrane fabrication yields larger heat conduction coefficients and a smaller temperature increase compared with those attained with the other membranes. As shown in Figure 6d, the fabrication method also affects the photomechanical response of the membranes, *i.e.*, the stress induced by laser irradiation. While both samples with CNP showed a five-fold increase in this response compared to pristine PDMS, the membranes with CHCl_3_ yielded the largest stress under laser irradiation perhaps due to the presence of larger carbon clusters. Therefore, albeit achieving a lower increase in temperature, the improved heat conduction in the CHCl_3_ membranes seems to favor the photomechanical response. Clearly, different effects related to the photothermal phenomenon can be achieved depending on the fabrication procedure. In what follows, we demonstrate a few examples showing potential applications for these laser driven membranes.

### 3.4. Applications

The PDMS/CNP blend can be applied and cured in different manners such as thin films, coatings, or even using mold replica techniques to obtain diverse geometries. In the following subsections, we will demonstrate a few devices created with this composite, showing the versatility of this photothermal material and its usefulness to provide light activated functionalities and novel alternatives for fabrication of optically reconfigurable devices.

#### 3.4.1. Coatings for Capillaries

Since the advent of soft lithography techniques, it has long been demonstrated that PDMS, together with other kinds of polymers, constitute hallmark materials for microfluidic applications [34,35]. One of the main reasons for this is because polymers can be easily molded and incorporated in a wide variety of microfluidic designs. Furthermore, these polymers can be used as matrices to host other types of materials such as CNP. This combination adds functionalities to these kinds of composites, allowing for the development of so-called smart micro-devices. PDMS/CNP composites can also be deposited along glass capillaries using fiber coating techniques, allowing for fabrication of photo-thermo-responsive devices capable of controlling thermocapillary flow [16]. This capability can lead to the realization of opto-pneumatic systems functioning as light-driven pistons for pumping fluids into microfluidic devices [17]. In this section, we demonstrate a new and straightforward application of PDMS/CNP coatings, used to generate two-phase boiling flows in micro-channels.

**Figure 7:**
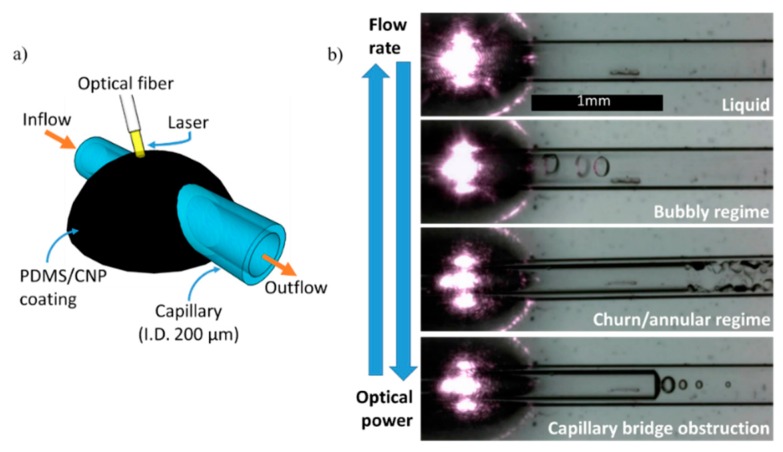
CNP/PDMS coatings for boiling flows: (**a**) schematic of the basic components to produce a boiling flow using PDMS+CNP coatings; (**b**) images showing the different flow regimes that can be achieved inside the capillary; the gas-liquid mixture can be varied by adjustments on the flow rate or the optical intensity of the laser diode. The bubbly flow regime constitutes the optimal flow condition since it allows good heat removal and optimal flow conditions. High optical powers or very low flow rates can create gas chambers or capillary bridges than can obstruct or introduce variations in the main flow.

Boiling flows in micro-channels has been an interesting subject because they have helped in understanding prototypes of cooling systems for micro-electronic circuits [36]. The basic operational idea of such systems can be stated as follows: consider a micro-channel with a flowing liquid intended to withdraw heat from a high temperature source. For a given configuration, an optimum liquid flow rate has to be obtained capable of providing maximum heat dissipation while simultaneously keeping optimal flow conditions in the micro-channel. At intermediate flow rates, heat is dissipated by the liquid but also by phase transitions, since part of the liquid evaporates in the form of micro-bubbles (heat removal is increased thanks to the latent heat of vaporization). Experimentally, we produced two-phase flows in a micro-channel by evaporating water inside a glass capillary (O.D. 330 µm, I.D. 200 µm) using a PDMS+CNP coating irradiated with laser light for heating (see Figure 7a). Figure 7b shows that different flow regimes can be produced, including the optimal bubbly flow regime. These were achieved upon adjusting the flow rate inside the capillary (up to 3.5 mL/h, or 30 mm/s) and the optical intensity at the output of the optical fiber (8 to 16 W/mm^2^). We are currently rationalizing these results in terms of two-phase boiling models and put this work in perspective with others that have employed different experimental setups.

#### 3.4.2. Coating for Fiber Optic Devices

Photothermal effects at the micrometric scale provide a useful tool for developing light-activated devices based on optical fibers. Coating of optical fibers has been shown to be a viable approach to generate light interaction with thin films of different materials. In particular, thin film deposition on optical fiber tips has been used to generate ultrasound probes [37,38]. Coatings of composite materials may also be deposited on intermediate sections of the fibers yielding light driven tunable devices [39]. Heat generation and detection in specific sections of an optical fiber is also of interest for optical sensors and their incorporation in multifunctional systems.

**Figure 8:**
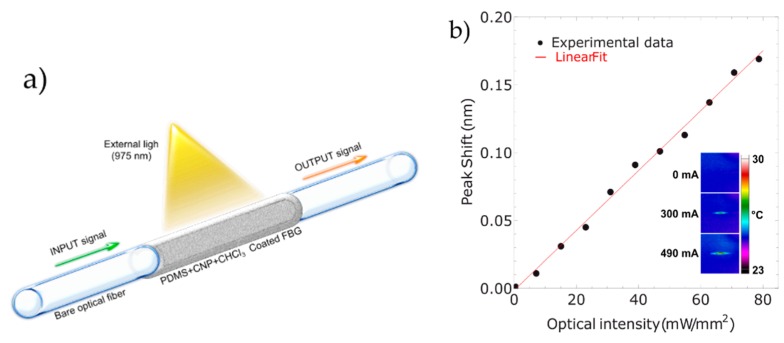
(**a**) schematic of FBG coated with a thin layer of the PDMS+CNP+CHCl_3_ composite irradiated with a focused IR laser beam; (**b**) FBG peak shift generated by light irradiation of the composite. The inset shows images obtained with the thermographic camera demonstrating localized heating on the irradiated section.

To demonstrate the potential of PDMS/CNP composites for realizing optically tunable fiber devices, a thin film coating (approximately 1.7 µm thick) of the PDMS+CNP+CHCl_3_ composite was applied on a fiber Bragg Grating (FBG) using the system described in [15]. After curing at 80 °C for 2 h, the coated FBG was irradiated with the 975 nm laser diode focused with a cylindrical lens to obtain uniform illumination over a length of 3 mm (see Figure 8a). The reflection peak of the FBG spectrum was initially centered at a wavelength of 1535.54 nm, and this was monitored with an optical spectrum analyzer as the output power of the laser diode was increased. As shown in Figure 8b, a maximum wavelength shift of approximately 170 pm was registered for the range of optical intensities used in this experiment. Using the typical value for the temperature sensitivity of FBGs (~10 nm/°C [40]), the resulting shift in peak wavelength is equivalent to a local increase in temperature of 16.8 °C. Heating of the FBG when irradiated with the laser was further confirmed with the thermographic camera (see inset in Figure 8b)**,** showing that the temperature is effectively increased only within the illuminated region. Hence, laser tunable FBGs can be attained with the proposed approach offering a novel means for realizing tunable fiber optic filters as an alternative to devices activated by electro-optic effects.

#### 3.4.3. Laser Micropatterning

As shown elsewhere, CNP-coated PDMS membranes may be etched using a low power, near infrared laser from a CD pickup head [12]. A motorized computer numerical control setup allows for tightly focusing the laser beam at the surface of the modified PDMS sample to ablate the desired micropatterns inside the material. This is achieved in a local fashion using laser-induced incandescence. However, as observed experimentally, this only happens when the CNPs coat the PDMS layer with a certain adhesion to the surface; otherwise, particles are immediately propelled away from the laser focal spot when light is shone either in CW or pulsed mode [12]. Features such as the dimensions of the obtained patterns depend strongly on the laser parameters. However, thickness, homogeneity and roughness of the CNP layer also play an important role.

In general, PDMS membranes with embedded CNP are desirable because flat surfaces yield improved resolution for the patterning process. However, for these membranes, our direct laser etching technique has proven to be inefficient, as no laser-induced incandescence, originating the ablation, had been obtained before. This is consistent with our findings since significant differences in heating and temperature increase are expected if the carbon nanoparticles are embedded inside the polymer or only coating the surface of the PDMS (see Section 3.1). When the CNP are embedded in PDMS, the generated heat is driven away from the focal spot and hence neither incandescence nor combustion occur.

**Figure 9:**
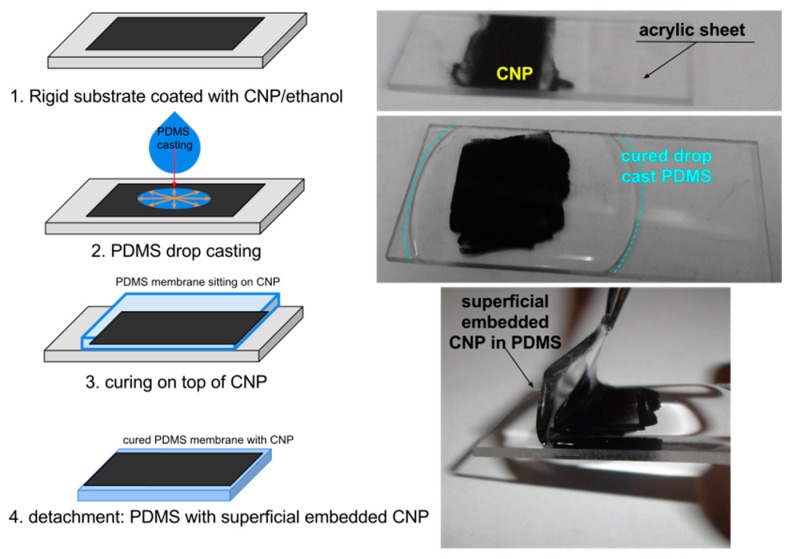
Adapted preparation method for superficially embedded CNPs in PDMS membranes.

Upon combining embedment and coating of CNP in PDMS membranes, we have finally been able to confirm that a thin, superficial but embedded layer would allow reaching high temperatures above the combustion threshold. Using a newly adapted simple method described in Figure 9, thin layers of carbon nanoparticles were embedded immediately underneath the surface of interest. A doctor blading technique was used to coat a flat surface of a clean, hard substrate (typically low roughness acrylic sheets or glass slide) with a thin layer of CNP in alcohol. Then, a small volume of PDMS was drop cast on this layer and cured immediately at 80 °C for 2 h. Spin coating may be used for thinner layers with similar results. As shown in Figure 9, after detaching the cured membrane, the surface in contact with the CNP layer replicated the flatness of the hard substrate and no additional roughness due to the CNP was observed on our new membranes.

**Figure 10:**
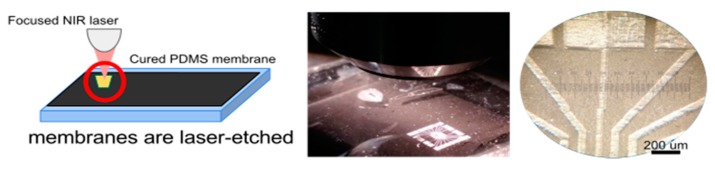
Example of a micropatterned design transferred to PDMS.

Unlike our previous PDMS/CNP samples, the process used to fabricate our new membranes allowed for etching the surface of the PDMS layer with improved resolution when compared with our previous results (see Figure 10). An optimal 2 µm wide spot was obtained in the best case, which is better than what was previously obtained with a CNP coating layer on PDMS [12]. This may be due to the quality of the PDMS surface, which leads to reducing scattering effects; furthermore, the absence of CNP roughness and reduction in homogeneity allow for concentrating more light, thereby obtaining highly localized thermal effects. Indeed, a much greater CNP homogeneity has been observed in these samples when compared to what is typically obtained with direct PDMS coating [12], thus leading to a greater in-plane resolution. Moreover, the laser ablation process is similar to that previously reported, offering the same control over all for the important laser parameters. It is interesting to remark that the depth of the ablated patterns is typically lower than what is obtained with direct PDMS/CNP coatings, reaching a maximum of 10 µm in the best case, as observed with a KLA-Tencor D600 profilometer. In addition, the optically absorbing layer is fully removed after etching, offering a great optical contrast between transparent and opaque areas for the development of optical masks, improving our previously reported results [41]. However, one disadvantage of this technique compared to that presented in [12] is that it is impossible to wash the residual CNP after the etching process; this limits its use for samples that do not require optical transparency around laser-etched micropatterns.

## 4. Conclusions

We have analyzed the photothermal effects produced in PDMS membranes with embedded carbon nanoparticles via light absorption. A simple heat conduction model along with experimental analysis was used to analyze the photothermal features of the membranes. As confirmed experimentally, heat generation and conduction in the membranes allow for obtaining different effects, such as photomechanical actuation and incandescence, that can be controlled depending on the physical and thermal properties of the composite material. The PDMS/CNP composites offer attractive features, such as ease of fabrication and processing, both of which are attractive for applications involving photothermal materials. In particular, we demonstrated that the composites may be used as membranes for laser micropatterning, or may be incorporated as coatings for microfluidic and fiber optic devices as new and simple alternatives to already existing technologies and fabrication methods. Variations when preparing the PDMS/CNP composites allow for achieving different photothermal features, which are closely related to carbon cluster distribution. This versatility makes these materials an attractive platform for developing light activated photonic and optofluidic devices.

## Figures and Tables

**Table 1:** Parameters used in Equation (8) to fit the experimental data shown in Figure 4.

Parameter	PDMS+CNP	PDMS+CNP+CHCl_3_	Notes
*L* (µm)	480	480	As determined from direct measurements.
*A* (mm^2^)	0.1465	0.1465	Estimated using the fiber tip-membrane separation distance.
β (mm^−1^)	4.4	2.8	Determined from experiments (see below).
*h*_1,2_ (W/(m^2^·K))	28	28	Typical value of heat convection in air.
*k* (W/(m·K))	0.26	0.6	Typical value for pristine PDMS is 0.1678 at 14.7 °C and 12,500 cst [24].
α (mm^2^/s)	0.18	0.42	Evaluated as α *= k/*ρ *Cp*, where *Cp* is the specific heat capacity and ρ is the materials density.
ℛ	0.04	0.04	Estimated considering a refractive index of 1.5
